# IGF1 Genetic Polymorphism and the Association between Vitamin D Status and BMI Percentiles in Children

**DOI:** 10.3390/children10101610

**Published:** 2023-09-27

**Authors:** Sigal Eilat-Adar, Eias Kassem, Mahmood Sindiani, Sigal Ben-Zaken

**Affiliations:** 1 Levinsky-Wingate Academic College, Wingate Campus, Netanya 4290200, Israelsigalbz@l-w.ac.il (S.B.-Z.); 2 Hillel-Yaffe Medical Center, Hadera 3810000, Israel; eiask@hy.health.gov.il

**Keywords:** IGF1 genetic polymorphism, vitamin D, BMI percentiles, children

## Abstract

Both the IGF1 axis and hypovitaminosis D play a role in childhood obesity, either as a cause or a causality. While some studies suggest an interrelation between vitamin D status, IGF1, and obesity, this mechanism remains obscure. The aim of this study, therefore, was to explore associations between four genetic polymorphisms in the IGF1 axis in hypovitaminosis D-related obesity. The study included 116 pre-pubertal Israeli Arab children (52 girls), mean age 9.4 ± 2.6. Serum 25(OH)D was measured and anthropometric measures were obtained. Genomic DNA was extracted from peripheral EDTA-treated anti-coagulated blood using a standard protocol. Genotypes were determined using the Taqman allelic discrimination assay. The IGF genetic score was computed according to the additive genetic score model. A moderate-to-high negative correlation (r = 0.580, p < 0.05) was seen between the vitamin D status and body mass index (BMI) percentile of participants with high GS. Yet, no correlations were seen between vitamin D status and BMI percentile for participants with a low-to-moderate genetic score (GS) (GS ≤ 2). These results suggest that IGF1 genetic scores associated with elevated circulating IGF1 may indicate a tendency toward developing hypovitaminosis D-associated obesity.

## 1. Introduction

Children suffering from obesity tend to exhibit normal-to-accelerated growth despite decreased growth hormone (GH) secretion. Associations between energy intake and body mass index (BMI)-Z score or childhood obesity have been identified [1,2]. Yet, conflicting reports exist as to the impact of obesity on components of the GH-insulin-like growth factor-I (IGFI)-IGF-Binding Proteins (IGFBPs) system [3]. The insulin-like growth factor (IGF) family of ligands, as well as binding proteins, constitute an important growth factor system in the development and maintenance of the body’s normal cell functioning. In addition, circulating IGFI concentrations are regulated by GH, that can be found in the body’s systemic circulation and expressed in tissue [4].

IGF1 is encoded by the *IGF1* gene that is located on chromosome 12 (12q23.2). Defects in this gene are known to cause a deficiency in the IGF polymorphism in the promotor region of the *IGFI* gene, which is associated with IGFI serum levels, birth weight, and body height in both adults [5,6,7] and children [8]. In addition, genetic polymorphisms in the promoter region of the IGFI gene have also been found to be associated with higher fat mass, body weight, and body mass index (BMI), as well as with a larger waist circumference in adolescents and children [8]. It should be noted, however, that such associations were not seen in children from a previous generation (even though they tended to be leaner than this present generation) [8].

Vitamin D deficiency is the most common type of vitamin deficiency in the world, and the worldwide prevalence of this condition varies greatly from country to country. Approximately one billion people worldwide are estimated to suffer from vitamin D insufficiency, with the largest prevalence at the pediatric age [9,10]. Vitamin D plays a role in many functions of the human body’s metabolism. The established health consequences of a vitamin D deficiency include increased fracture risk, tooth loss, osteomalacia, and rickets [11], as well as impact on cognitive function [12], and inflammatory bowel disease among children and adolescents [13] Therefore, identifying and treating vitamin D deficiency during this period may be of particular importance.

The vitamin D precursor 7-dehydrocholesterol, found primarily in the epidermal layer of the skin, is activated by sunlight to produce vitamin D-3 and is bound to the vitamin D binding protein. This complex is then transported to the liver where it is rapidly hydroxylated by vitamin D-25-hydroxylase to form 25-hydroxyvitamin D [25(OH)D]—the major circulating form of vitamin D [11]. Through further hydroxylation by the enzyme 25-hydroxyvitamin D-1-α-hydroxylase, 25(OH)D is converted into the biologically active form of 1,25 di-hydroxyvitamin D [1,25(OH)2D] which regulates more than 200 genes, directly or indirectly, by binding them to vitamin D nuclear hormone receptors (VDRs) [14,15].

Studies suggest a possible interaction between vitamin D and IGF1 [16,17,18]. Reductions in circulating IGF1 and in some IGF1 binding proteins (especially IGFBP-1), as well as hypovitaminosis D, have been found to be associated with metabolic syndrome and its related components. In obesity, both IGF1 concentrations and vitamin D status are reduced [19,20]; yet, for both variables, associations have been seen between lower concentrations and high blood pressure, disturbed glucose metabolism, cardiovascular disease, and adverse lipid profiles, regardless of body mass [21]. Associations between metabolic risk and vitamin D and IGF1 axes have been seen in clinical trials, prospective studies, and dose-related effects, indicating that these associations might be causal. Still, the mechanism through which vitamin D and IGF1 axes lead to human disease are not yet fully understood. Moreover, regardless of evidence indicating physiological interactions between these risk factors [22,23,24,25], much is still unknown about their combined impact, especially in children and adolescents. As such, the objective of this study was to examine the associations between IGFI genetic variant vitamin D status and various anthropometric measures among pre-pubertal children.

## 2. Materials and Methods

### 2.1. Study Population

The participants in this study included 116 Arab children from Israel, all pre-pubertal (Tanner stage 1–2), including 52 girls, ages 6–12 (average ± standard deviation (AVG (SD)) age 9.4 ± 2.6). Their body measurements were documented and blood was taken to assess their vitamin D status. Approval to conduct this study was received by the Institutional Review Board of the Hillel Yaffe Medical Center, om Hadera, Israel, as per the Declaration of Helsinki (Approval # HYMC 0018-12). All participants and their parents signed an informed consent form for participation in this study.

### 2.2. Vitamin D Status

To measure Serum 25(OH)D, a chemiluminescent immunoassay was used (ARUP Laboratories, Salt Lake City, UT, USA). The intra-assay variation coefficient at 30.8 nmol/liter was 6% and was 4% at 90.5 nmol/liter; the inter-assay variation coefficient at 31.0 nmol/liter was 8% and was 6% at 90.5 nmol/liter. Finally, vitamin D status was defined as deficient (serum 25(OH)D ≤ 20 ng/mL), insufficient (serum 25(OH)D > 20 to <30 ng/mL), or sufficient (serum 25(OH)D ≥ 30 ng/mL) [26,27].

### 2.3. Anthropometric Measures

To measure the participants’ weight and height, standard calibrated scales and stadiometers were used; their BMI was then calculated based on these measurements. Obese or overweight participants were determined based on percentiles for age, as per the standards stated by the National Center for Health Statistics and the Centers for Disease Control [28]. Participants were classified as overweight if their BMI was above the 85th percentile but below the 95th percentile for their given age group; they were classified as obese if their BMI was above the 95th percentile for their given age group. Finally, the participants’ physical maturity was determined through a widely-used self-administered questionnaire as a noninvasive indicator of their pubertal status [29,30].

### 2.4. Genotyping

Using standard protocols, genomic DNA was extracted from peripheral Ethylenediaminetetraacetic acid (EDTA)-treated anti-coagulated blood. The genotype analysis was then performed in the Genetics and Molecular Biology Laboratory at the Wingate Institute (Academic College of Physical Education and Sport Sciences). The internal control was ensured for each genotype analysis using negative and positive controls from different DNA aliquots that had already been genotyped using the same method, in line with recent guidelines for replicating genotype–phenotype chi-square association research studies [31].

Genotypes of the examined genetic polymorphisms (*IGF1*-C1245T rs35767; *IGF1* T/C rs6220; *IGF1* A/G rs7136446; and *IGF1R* A/C rs1464430) were defined based on the TaqMan allelic discrimination assay. To set up TaqMan allelic discrimination essay primers and probes, the Assay-by-Design service (https://www.thermofisher.com/il/en/home.html, accessed on 1 December 2021) was utilized (see Table 1).

The Polymerase Chain Reaction (PCR) mixture comprised of 5 ng genomic DNA (1 ng/μL), 0.125 μL TaqMan assay (40*, ABI, 0.025 μL/μL), 2.5 μL Master mix (ABI) (0.5 μL/μL), and 2.375 μL water. PCR was conducted with 96 well PCR plates in an ABI 7300 PCR system (Applied Biosystems Inc., Foster City, CA, USA), with an initial denaturation of 5 min at 95 °C, followed by 40 cycles with a denaturation of 15 s at 95 °C. This was performed in addition to 60s annealing and extension at 63 °C. The results were then analyzed via the ABI TaqMan 7900HT, based on the sequence detection system 2.22 software (Applied Biosystems Inc., Foster City, CA, USA).

### 2.5. Genetic Score

The combined impact of four IGF axis polymorphisms was computed as the IGF Genetic Score (IGF-GS), based on a previously used model [32]. To do so, each genotype was scored within each polymorphism, according to the genotype’s related vitamin D status (i.e., the BMI percentile correlation). An additive model was assumed, as per the number of alleles associated with the vitamin D status, i.e., the BMI percentile correlation that was conducted for each participant’s polymorphism. In this manner, a genotype score (GS) of 0, 1, or 2 was assigned to each genotype, each being theoretically associated with a low, medium, or high vitamin D status–BMI percentile correlation, as seen in Table 2. The studied polymorphisms are all listed in Table 3. The IGF-GS score scale ranged from 0 (the “worst” GS, which represents low genetic potential towards vitamin D status–BMI percentile correlation) to 7 (the “best” GS, which represents high genetic potential towards vitamin D status–BMI percentile correlations).

### 2.6. Statistical Analysis

The achieved data are presented as mean ± standard deviation (SD) for continuous variables and n (%) for categorical ones. *t*-tests were used for comparison between continuous variables, while chi-square was calculated for categorical variables. Finally, correlations between the participants’ anthropometric measures and their vitamin D status were conducted using Pearson correlations for normal distribution. All data analyses were conducted using SPSS version 20.5.

## 3. Results

Table 4 presents the participants’ anthropometric measurements and their vitamin D status. Continuous variables are presented as (AVG (SD)). Categorical variables are presented as n (%). All participants were found to be vitamin D insufficient (i.e., below 25 ngr/mL), with 80% fitting the deficient category. In addition, more than 45% of the children were classified as overweight or obese.

Table 5 presents the data on allele and genotype frequencies. Our findings indicate that the genotype subtype did not differ by sex or age. Moreover, the genotype distribution was found to be in agreement with the Hardy–Weinberg equilibrium in overweight/obese children and participants of normal weight (*p* > 0.05) for all studied genetic polymorphisms. Genotype and allele frequencies did not differ between normal-weight and overweight/obese children for *IGF1*-C1245T (rs35767), *IGF1* T/C (rs6220), and *IGF1R* A/G (rs1464430) polymorphisms. The prevalence of *IGF1* rs7136446 AA genotype carriers was found to be higher in the normal-weight children (32.4%) than in the overweight/obese participants (12.5%) (*p* = 0.019).

Due to the low prevalence of *IGF1* rs3567 TT genotype carriers, we studied the prevalence of CT and TT genotype carriers together. A negative, low-to-moderate correlation between vitamin D status and BMI percentile was seen for *IGF1* rs3567 T allele carriers, *IGF1* rs1464430 C allele carriers, and *IGF1* rs6220 TT genotype carriers. These correlations were close to statistical significance (*p* = 0.060, 0.080, and 0.081 for *IGF1* rs6220, *IGF1* rs1464430, and *IGF1* rs6220, respectively).

**Figure 1:**
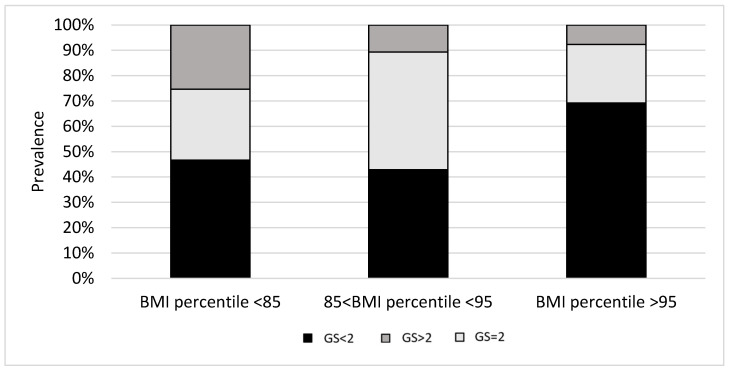
IGF1 GS prevalence according to BMI percentile.

Participants’ GS according to their weight category are presented in Figure 1. In total, 56 participants had low GS (<2), 37 had moderate GS (=2), and 23 had high GS (>2). A high prevalence (69%) of participants with low GS were found among the obese participants (BMI percentile > 95%) compared to overweight (85% < BMI percentile < 95%) and normal-weight (BMI percentile < 85%) participants (43% and 47% for overweight and normal-weight children, respectively), yet this difference was not statistically significant. A high prevalence of high GS was found among normal-weight participants compared to overweight and obese participants, yet this difference was not statistically significant either.

**Figure 2:**
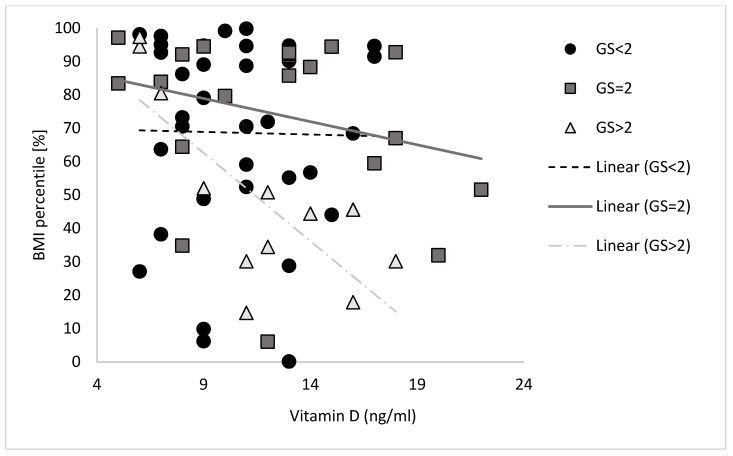
Vitamin D status and BMI percentile according to IGF1 GS percentile.

Correlations between the vitamin D status and the BMI percentile according to the GS are presented in Figure 2. No correlation was found between the vitamin D status and the BMI percentile for participants with a low or moderate GS score (GS ≤ 2). A moderate to high negative correlation (r = 0.580, *p* < 0.05) between the vitamin D status and the BMI percentile was found for participants with high GS.

## 4. Discussion

The main finding of the current study is that IGF1 GS may mediate the relationship between a person’s vitamin D status and their BMI percentile. Participants carrying high IGF1 GS exhibited a significant inverse relationship between their vitamin D status and their BMI percentile, whereby the participants’ elevated levels of vitamin D were associated with a low BMI percentile, but not among participants carrying low IGF1 GS measures. As such, the results suggest that participants carrying genotypes associated with elevated circulating IGF1 are prone to developing hypovitaminosis D-associated obesity.

The IGF axis genetic panel was comprised of four polymorphisms which have been found to be associated with circulating IGF1 levels. The C allele of *IGF-1* T/C (rs6220) polymorphism, for example, is associated with higher levels of circulating IGF-1 [33]; additionally, the *IGF1* C1245T polymorphism (rs35767) is associated with higher levels of circulating IGF1 [34,35,36,37,38,39]; finally, the G allele of a different *IGF1* gene polymorphism (rs7136446) has been found to be associated with higher circulating IGF-1 [35]. Free IGF-I levels are inversely correlated with a range of obesity and body composition measures [40,41]. In the current study, a strong prevalence of high IGF scores associated with increased levels of circulating IGF1 was seen among participants with a BMI percentile lower than 85%; however, a high prevalence of low IGF scores, which is correlated with lower levels of circulating IGF1, was found in children with a higher than 95% BMI percentile.

Our results suggest that among participants with higher GS (associated with elevated circulating IGF1), those who exhibit lower levels of vitamin D (hypovitaminosis D) exhibit higher BMI percentiles. This means that high GS (and possibly elevated circulating IGF1) combined with low levels of vitamin D could serve as a risk factor for obesity, especially as the association between hypovitaminosis D and obesity is well established [42,43]. In addition, endocrine disorders, such as altered somatotropic axis, are common in obesity. The insulin-like growth factor-1 (IGF1) values, however, are controversial. It is also yet to be determined whether Vitamin D increases circulating IGF1 [44] or vice versa; IGF1 causes an increase in the circulating levels of vitamin D by stimulating the expression and activity of the 1α-hydoxylase that produces 1,25(OH)2D in the kidney [45,46]. However, when vitamin D levels decrease (if IGF1 fails to increase vitamin D level or due to other causes) hypovitaminosis D-associated obesity might occur [47].

Obesity in childhood causes a wide range of serious complications and increases the risk of premature morbidity and mortality [48]. The primary common phenotype of obesity is fat accumulation in adipose tissue and other organs. Increased adiposity is strongly related to GH, insulin-like growth factor-I (IGFI) axis dysfunction [49] GH. A mixture of peptides, may play a major role in controlling longitudinal growth in children [50]. GH is bound to the circulating GH binding proteins (GHBP) [51] and exerts its biological effects directly on target cells by binding to cell membrane receptors or/and through IGFI [52]. The mechanism responsible for the altered GH and IGF1 secretion observed in obesity is largely unclear. Since GH has a lipolytic effect in both obese and normal-weight participants, it is suggested that low GH levels blunt lipolysis in obese subjects, thereby contributing to increased fat development [53]. Moreover, free IGFI levels have been found to inversely correlate with different measures of obesity and body composition [53], while obesity is considered to be a risk factor for developing hypovitaminosis D [54].

Different mechanisms have been hypothesized to explain the association between hypovitaminosis D and obesity, including lower dietary intake of vitamin D, less skin exposure to sunlight due to less outdoor physical activity, decreased intestinal absorption, impaired hydroxylation in adipose tissue, and 25(OH)D accumulation in fat [55]. In a systematic review and meta-analysis of 10 intervention trials, a high dose of vitamin D supplementation (over 4000 IU/d) did not alter the BMI status in children [56]. However, it has also been speculated that a vitamin D deficiency itself could cause obesity; the fat-solubility of vitamin D has led to the hypothesis whereby a sequestration process occurs in body fat depots resulting in a lower bioavailability in the obese state [57]. Whether vitamin D is a cause or consequence of obesity is still unclear [58]. It is possible that IGF1 polymorphism is one of the reasons for the inconsistent results of vitamin D supplementation and weight loss.

Numerous studies have hypothesized an interaction between vitamin D and GH/IGF-1. However, the exact mechanism by which they influence one another remains unknown [59], particularly in obesity. Reductions in circulating IGF1 and hypovitaminosis D, and a range of IGF1 binding proteins, have been found to be associated with metabolic syndrome and its components. Both vitamin D status and IGF1 concentrations are decreased in obese individuals [19,20,60]. Despite evidence for a physiological interaction between vitamin D and IGF1 axes with metabolic risk [22,23,24], little is known about their joint effects.

Studies indicate interrelations between vitamin D and IGF1 axes. For instance, the anticancer impact of higher vitamin D availability includes the promoting of antiproliferative effects on different body tissues, through increased IGFBPs production combined with the suppression of cell growth-promoting IGFBP2 [61]. As such, IGF-1 could play an impactful role through changes to vitamin D activation, whereas 1,25(OH)2D may act partly through changes to IGF1 axis regulation.

Our results suggest a possible mechanism for the interrelations between IGF1, vitamin D, and obesity. Participants who are genetically predisposed toward elevated levels of circulating IGF1 are prone to a low BMI percentile, especially if their vitamin D status is elevated. Yet, childhood obesity is a multifactorial trait resulting from various factors with complicated interactions. Whether obesity is the cause or the result of hypovitaminosis D is still to be determined.

## 5. Limitations

Despite the contribution of this study to the literature, this study has several limitations. First, although the sample size is relatively small, we decided to include two genetic polymorphisms in the GS scores that were close to statistical significance. Moreover, children are seriously under-investigated, and this exploratory study should be followed by larger sample size studies and more genetic polymorphisms. Finally, other environmental factors, such as nutritional intake, physical activity, and sun exposure were not measured. Future studies focusing on sex differences and physical and nutritional effects are suggested.

## Figures and Tables

**Table 1:** Primers and probes for studied polymorphisms.

Primer Sequences:		Probe Sequences:	
Forward	Reverse	Forward: VIC	Reverse: FAM
*IGF1* A/G (rs7136446), NC_000012.12			
AATTGGTTACCTGCTACATTGA	GGAGTTAACGCATCTCCTTACTG	CGCGTAGTCGAGCG	CGCTCGCTGCCCTAAGTGCT
*IGF1*-C1245T (rs35767), NC_000023.11			
GGATTTCAAGCAGAACTGTGTTTTCA	GGTGGAAATAACCTGGACCTTGAAT	TTTTTCCGCATGACTCT	TTTTTTTTCCACATGCTCT
*IGF1* T/C (rs6220)			
AACAAAGAGATTTCTACCAGTGAAAGG	GCCTAGAAAAGAAGGAATCATTGT	AGTAAAACCTTGTTT AATAC	AGTAAAACCTCGTTT AATA
*IGF1R* A/G (rs1464430)			
GGATTTCAAGCAGAACTGTGTTTTCA	GGTGGAAATAACCTGGACCTTGAAT	TTTTTTCCGCATGACTCT	TTTTTTTTCCACATGACTCT

**Table 2:** Current study: vitamin D status–BMI percentile correlations according to genotype.

IGF1_rs3567	CC			CT + TT
N	81			33
r, *p*				−0.331, 0.060
**IGF1_rs7136446**	AA	AG	GG	AG + GG
N	29	58	27	85
r, *p*	−0.641, 0.014			
**IGF1R_rs1464430**	AA	AC	CC	AC + CC
N	30	62	22	84
r, *p*				−0.244, 0.081
**IGF1_rs6220**	TT	TC	CC	TC + CC
N	44	51	19	70
r, *p*	−0.337, 0.080			

N = number of genotype carriers; r = correlation ratio; *p* = statistical significance.

**Table 3:** Genetic scoring of IGF-1 axis genetic polymorphisms based on genotypes’ association to vitamin D status.

Symbol	Polymorphism	MAF	Genetic Score (GS)
IGF1	A/G (rs7136446)	28%	AA = 2, AG = 0, GG = 0
	-C1245T (rs3567)	30%	CC = 0, CT = 0, TT = 1
	T/C (rs6220)	36%	TT = 1, TC = 0, CC = 0
IGF1R	A/G (rs1464430)	40%	AA = 0, AC = 0, CC = 1

MAF—Minor allele frequency (from https://www.genecards.org/, accessed on 1 August 2021).

**Table 4:** Participants’ anthropometric measures and vitamin D status.

	(AVG (SD)) or n (%)
Age	9.4 (2.6)
Mean BMI percentile	65.0 (29.3)
Mean Body Fat	16.9 (5.7)
Overweight prevalence	38 (33.3)
Obesity prevalence	15 (13.1)
Height percentile	54.2 (35.3)
Weight percentile	63.2 (31.2)
Serum vitamin D (mcg/dL)	11.2 (3.9)
Borderline vitamin D (%)	(19.4)
Vitamin D deficiency (%)	(80.6)

**Table 5:** Obesity/OW according to IGF polymorphism.

	Total	Normal Weight	Overweight/Obese	*p*
IGF1 rs7136446 A/G				
AA	29 (25.4)	24 (32.4)	5 (12.5)	0.052
AG	58 (50.9)	34 (45.9)	24 (60.0)	
GG	27 (23.7)	16 (21.6)	11 (27.5)	
A—allele	116 (50.9)	82 (54.7)	34 (42.5)	0.079
G—allele	112 (49.3)	68 (45.3)	46 (57.5)	
IGF1 rs1464430 A/C				
AA	30 (26.3)	22 (29.7)	8 (20.0)	0.216
AC	62 (54.4)	41 (55.4)	21 (52.5)	
CC	22 (19.3)	11 (14.9)	11 (27.5)	
A—allele	122 (53.5)	85 (57.4)	37 (46.3)	0.106
C—allele	106 (46.5)	63 (42.6)	43 (53.7)	
IGF1 rs3567 C/T				
CC	81 (71.0)	51 (68.9)	30 (75.0)	0.231
CT	32 (28.1)	23 (31.1)	9 (22.5)	
TT	1 (0.9)	0 (0)	1 (0.9)	
C—allele	194 (85.1)	125 (84.4)	69 (86.2)	0.717
T—allele	34 (14.9)	23 (15.6)	11 (13.8)	
IGF1 rs6220 T/C				
TT	44 (38.6)	30 (40.5)	14 (35.0)	0.708
TC	51 (44.7)	31 (41.9)	20 (50.0)	
CC	19 (16.7)	13 (17.6)	6 (15.0)	
T—allele	139 (60.9)	91 (61.5)	48 (60.0)	0.827
C—allele	89 (39.1)	57 (38.5)	32 (40.0)	

Notes: χ^2^ = 5.439 (df = 1), *p* = 0.0197 for IGF1 rs7136446 AA carriers’ prevalence among normal-weight participants compared to overweight/obese children (above 85th BMI percentile for age).

## Data Availability

The data presented in this study are available on request from the corresponding author.
